# Lethal Sudan virus infection in IFNAR^-/-^ mice is characterized by key inflammatory features of filovirus disease

**DOI:** 10.1371/journal.pone.0347080

**Published:** 2026-09-29

**Authors:** Michelle Gellhorn Serra, Cornelius Rohde, Lucie Sauerhering, Lars Meier, Lennart Kämper, Pauline Neubecker, Markus Eickmann, Alexandra Kupke, Stephan Becker, Anke-Dorothee Werner

**Affiliations:** 1 Institute of Virology, Marburg University, Marburg, Germany; 2 German Center for Infection Research (DZIF), Partner Site Gießen-Marburg-Langen, Marburg, Germany; University of Nebraska-Lincoln, UNITED STATES OF AMERICA

## Abstract

Sudan virus (SUDV) is a member of the family *Filoviridae*, which comprises highly pathogenic viruses associated with unusually high case fatality rates. The development of medical countermeasures against filoviruses, including antivirals, vaccines, and therapeutic antibodies, requires preclinical evaluation in suitable animal models. C57BL/6J IFNAR^-/-^ mice, which lack the type I interferon (IFN-α/β) receptor, have been reported to be susceptible to filovirus infections, although their impaired innate immune response may represent a potential limitation of the model. Here, we characterized the clinical, virological, pathological, and inflammatory responses following SUDV Boniface infection in IFNAR^-/-^ mice. Following infection, animals developed progressive clinical disease characterized by substantial weight loss and pronounced changes in behavior and appearance. Mice reached the predefined clinical endpoint 3–5 days post infection. *Post mortem* analysis of terminal samples revealed high viral loads and viral genome copies in all tested organs as well as in serum, consistent with widespread systemic dissemination. Histopathological examination revealed prominent lesions, particularly in the liver and spleen. In addition, terminal serum analysis revealed a broad and pronounced inflammatory response, with significantly elevated concentrations of multiple cytokines and chemokines compared with non-infected controls, encompassing pro-inflammatory, regulatory, and hematopoietic mediators. Together, these findings characterize the clinical course, systemic viral dissemination, tissue pathology, and terminal inflammatory response associated with lethal SUDV infection in IFNAR^-/-^ mice. The observed cytokine and chemokine elevations are consistent with inflammatory responses described in filovirus infections, although the single terminal sampling time point and limited cohort size preclude conclusions regarding the temporal development or mechanistic basis of this response. These data support the utility of this model for investigating filovirus pathogenesis and infection-associated immune responses and warrant further evaluation of its suitability for SUDV infection and for preclinical studies of filovirus countermeasures.

## Introduction

The Sudan virus (SUDV) belongs to the order *Mononegavirales* and is a member of the genus *Orthoebolavirus* within the *Filoviridae* family. Since SUDV was first reported in 1976, the virus has periodically emerged, causing nine additional outbreaks with case fatality rates ranging between 41 and 70% [1,2]. SUDV and other filoviruses are listed as priority pathogens by the WHO R&D Blue-print initiative as they pose a great threat to public health care systems because of their epidemic potential and insufficient medical countermeasures [3]. Most filoviruses are therefore classified as biosafety level-4 (BSL-4) pathogens.

Wild-type (WT) rodents are not susceptible to filoviruses and show minimal or no symptoms. After adaptation through serial passaging in rodents, filoviruses adopt genomic mutations which are accompanied by the development of severe disease [4–6]. As a result, these adapted viruses may not accurately reflect WT pathogenesis and vaccines, antibodies or small molecule antivirals developed against adapted viruses might not be optimal to counteract WT virus infection. Immunocompromised rodents therefore represent a useful tool that supports WT filovirus infection efficiently [4,7,8]. Common rodent models include the interferon-α/β receptor knockout mice (IFNAR^-/-^), the double interferon-α/β and γ receptor knockout (IFNAGR^-/-^), interferon-γ receptor knockout (IFNGR^-/-^), the cytoplasmic signal transducer and activator of transcription-1 protein knockout (STAT-1^-/-^) or the severe combined immunodeficiency (SCID) mice. Their inherently compromised immune responses represent limitations regarding infection and disease progression, as detailed information on the mounted immune response in successfully infected animals is missing for a number of mouse and virus strain combinations. Nevertheless, IFNAR^-/-^ mice, for instance, can efficiently mount humoral and cellular immune responses, and are therefore commonly used to evaluate different vaccine strategies, including those for Zika virus [9], Ebola virus [8,10,11] and Crimean-Congo haemorrhagic fever virus [12]. For SUDV, several of such immunocompromised mouse models exist with different combinations of mice and virus strains [4,7,8,13–17]. The lethality of SUDV infection in these models varies and the majority of these studies focused on haematology and serum chemistry. This variability is likely driven by differences in viral isolates, experimental conditions, and humane endpoint criteria, which together influence the observed disease severity and lethality.

In the present study, we have characterized cytokine and chemokine responses in an IFNAR^-/-^ mouse model of SUDV Boniface infection, which share similarities with other animal models and human infection.

## Methods

### Cells and viruses

SUDV isolate Boniface was kindly provided by the Bernhard-Nocht-Institut, Hamburg, Germany. This isolate originated from the 1976 outbreak and was cultured and titrated using Vero C1008 cells (clone E6, VeroE6, ATCC CRL-1586, African green monkey kidney cell), cultivated in Dulbecco's modified Eagle medium (DMEM) supplemented with 3% fetal bovine serum, 1% L-Glutamine (200 mM), and 1% penicillin (50 units/ml) and streptomycin (50 mg/ml) (DMEM ++) at 37 °C and 5% CO_2_. Viral titers were calculated based on TCID_50_ and plaque assays (using VeroE6 cells). The final virus stock was sequenced and tested negative for contaminations such as mycoplasma or other virus infections. Passage x + 4 was used for the challenge

All experiments with SUDV were performed in the high containment facility (BSL-4) of the Institute for Virology, University of Marburg, Germany, according to national and international regulations.

### Mouse experiments

Interferon receptor alpha/beta knockout mice (IFNAR^-/-^) mice [18] with a C57BL/6J background were selected because of their reportedly high susceptibility for filovirus infections [4,8] and were generously provided by U. Kalinke (Twincore, Hannover). Mice of both sexes were obtained from our in-house breeding colony. All experiments and protocols were approved by the regional authorities *Regierungspräsidium Gießen* AZ V54 –19 c 20 15 h 01 MR 20/7 Nr. G 64/2024), conducted according to the recommendations of Federation of European Laboratory Animal Science Associations and *Gesellschaft für Versuchstierkunde* (Society for Laboratory Animal Science, GV-SOLAS) and in compliance with the German animal welfare act and Directive 2010/63/EU. All procedures were carried out by personnel who had received appropriate training and certification for working with mice.

Mice were housed as described previously [12,19]. Three female (F) and three male mice (M), aged 6 months, were briefly anesthetized using isoflurane (CP-Pharma, Burgdorf, Germany) and infected intraperitoneally (i.p.) with approx. 1,000 plaque forming units (PFU) of SUDV in DMEM at a total volume of 200 µL. Back-titration of the inoculum confirmed a dose of 870 PFU. Mice were checked daily for changes in appearance, behaviour or body weight. Blood samples were drawn from the facial vein 3 days post infection (dpi) and immediately prior to euthanasia. Upon reaching the humane endpoints (clinical score of 10 or higher or score of 6 on two consecutive days; see S1 Table for more detailed information), the mice were euthanized by cervical dislocation under isoflurane anaesthesia.

Tissue samples from mesenteric lymph nodes, spleen, liver, kidney, lung, thymus, eye and brain as well as ovaries or testicles and accessory genital glands were collected and used for downstream *post mortem* analyses.

### Determination of viral loads and titers

Tissue samples were homogenized in 1 mL DMEM with ceramic and glass beads (Lysing Matrix H 2-mL tubes, MP Biomedicals) in a Mixer Mill MM 400 (Retsch, Germany) for 5 min. Afterwards, the homogenates were centrifuged for 5 min at 2,400 rpm and the supernatant was used for the isolation and quantification of viral RNA using qRT-PCR and the determination of viral titers in the different organs via TCID_50_ method.

### qRT-PCR of serum and tissues

Isolation and quantification of viral RNA was performed as previously described [20]. Briefly, viral RNA was isolated using the RNeasy Mini Kit (Qiagen) according to the manufacturer’s instructions and purified using the Quick-DNA/RNA viral MagBead kit (Zymo research) according to manufacturer's instructions along with the fully automatic nucleic acid extraction system (Tecan). Viral loads were assessed using the RealStar® Filovirus Screen RT-PCR Kit 1.0 (altona Diagnostics) according to manufacturer's instructions (including the following adaptation: volumes for both master mix and RNA were halved) on a qTOWER (Analytik Jena).

### Virus titration by TCID_50_

To determine the levels of infectious virus in organ homogenates or serum samples, VeroE6 cells were seeded in 96-well plates (1x 10^4^ cells/well) and infected with 10-fold serial dilutions of supernatants from either organ homogenates or serum samples. At 6 dpi the cytopathic effect (CPE) was analyzed and TCID_50_/ml were calculated according to Spearman and Kerber [21]. The limit of detection (LOD) for the organs was calculated by using the minimal detectable TCID₅₀/ml titer possible and relating it to the heaviest organ sample measured. This approach yields the lowest possible detectable value. The result was then extrapolated to 25 mg of tissue, providing the final LOD expressed as TCID₅₀ per 25 mg of tissue.

### Histopathological examination

The histological analysis was performed as described previously [22,23]. Briefly, collected organs were fixed in formalin and embedded in paraffin. Sections with a 4 µM thickness were cut with a microtome and stained with haematoxylin and eosin (H&E) for histopathological analysis. Furthermore, SUDV-specific RNA was stained by *in situ* hybridization (ISH) using the RNAscope^®^ 2.5 HD Assay—RED Kit (Cat. No. 322360) from Bio-Techne, with the probes V-SudanEbola-NP-sense (Cat No. 479281) and a custom designed probe V-SudanEbola-NP-O1-C1 (1846971-C1). The V-SudanEbola-NP-sense probe, which was used throughout the study, is directed against SUDV Gulu strain and detects antisense (antigenomic) RNA. To assess whether potential differences in staining patterns were attributable to strain-specific sequence variation, a custom probe, V-SudanEbola-NP-O1-C1, targeting the SUDV Boniface strain, was additionally designed and tested. This custom probe was used solely as a control for comparison of staining patterns and was not employed for routine analyses. Signal amplification was then performed using alkaline-phosphatase–labelled probes in combination with Fast Red substrate, which allowed signal detection. In parallel, a Negative Control Probe (Cat No. 310043) was used to control for background staining. Finally, the slides were counterstained with Gill’s Hematoxylin I and 0.02% ammonia water. Histological samples were evaluated independently and semi quantitatively by a trained scientist and a blinded veterinary pathologist. For liver tissue, the following parameters were assessed: necrotic foci, immune cell infiltration, steatosis, eosinophilic liver cells, and structural tissue damage. For the spleen following parameters were evaluated: white pulp atrophy (lymphoid depletion), congestion, focal single-cell necrosis, and infiltration by polymorphonuclear cells. Each parameter was scored using a 0–3 scale, with higher scores indicating greater severity.

### Multiplex ELISA – Luminex technology

To analyze inflammatory responses, final serum samples were screened for the release of cytokines and chemokines using the Bio-Plex Mouse Cytokine 23-Plex Assay by BioRad, as previously described [24]. Briefly, magnetic beads, each containing a unique mixture of red and infrared fluorophores for identification and coupled to specific antibodies for analyte detection, were transferred to the assay plate and washed twice. Then, standards, samples, and controls were added and incubated for 30 min, followed by 25 µl detection antibody solution incubated for 30 min and 50 µl streptavidin-phycoerythrin (PE) for 10 min. Finally, the samples were resuspended in 125 µl assay buffer and mixed for 30 s. Data were acquired on a BioPlex 200 system (BioRad Laboratories). The incubation steps were performed on a shaker at 850 r.p.m. and room temperature (RT). Washing steps were performed after each incubation step on a Bio-Plex Pro Wash Station (BioRad Laboratories). The analyzed cytokines included IL-1α, IL-1β, IL-2, IL-3 IL-4, IL-5, IL-6, IL-9, IL-10, IL-12 (p40), IL-12 (p70), IL-13, IL-17A, eotaxin, IFN-γ, TNF-α, KC, RANTES, MIP-1α, MIP-1β, MCP-1, G-CSF and GM-CSF (see S2 Table). A five-parameter logistic regression was used to calculate the concentrations of the different analytes in the samples. The lower and upper limit of quantification were defined as the lowest and highest standards for each analyte, respectively. Serum samples of historical, non-infected C57BL/6J IFNAR^-/-^ mice were used as controls. Control animals were age-matched and included both sexes, comparable to the infected cohort. To minimize inter-assay variability, serum samples from infected and control animals were analyzed in parallel within the same assay run using the same Luminex panel. For statistical analysis, mediator concentrations were log-transformed, and differences between SUDV-infected mice and uninfected control mice were assessed for each analyte using an unpaired t-test using Graphpad Prism 8.0.2 software.

### Next generation sequencing

Libraries for Illumina next generation sequencing of SUDV Boniface were prepared using the Twist Total Nucleic Acids Library Preparation EF Kit 2.0 (Twist Bioscience) for Viral Pathogen Detection and Characterization, as well as the Twist Target Enrichment Protocol (Rev. 2.0) with the Comprehensive Viral Research Panel. Libraries were sequenced on an Illumina iSeq 100 system, paired end reads 2 x 151 bp. Quality- and adapter trimming of raw reads, mapping and consensus sequence generation were performed using Geneious Prime 2022 build-in tools (BBduk, Geneious mapper, consensus sequence generation highest quality with cutoff 60%, respectively). Detected variants were visually validated in the alignment view. Detailed sequencing strategy and primers are available on request. Sequencing revealed three mutations (g605a (NP E150K), t5061c (VP40 L203P) and c12750t (L, silent mutation) compared to the reference sequence (FJ968794).

## Results

### Sudan virus infection causes a uniformly lethal disease in IFNAR^-/-^ mice

3 female and 3 male IFNAR^-/-^ mice were infected intraperitoneally (i.p.) with approx. 1,000 PFU of SUDV Boniface isolate. Disease progression was monitored by measuring body weight and determining the overall appearance and behaviour daily (Fig 1A). Each clinical symptom was assessed on a scale from 0 to 10 with 10 representing the most severe. The additive clinical score summarized the results of the different parameters analyzed daily (Fig 1B and 1C). Animals were euthanized when the summed clinical score reached values of ≥10 on a single day or ≥6 on two consecutive days.

**Fig 1:**
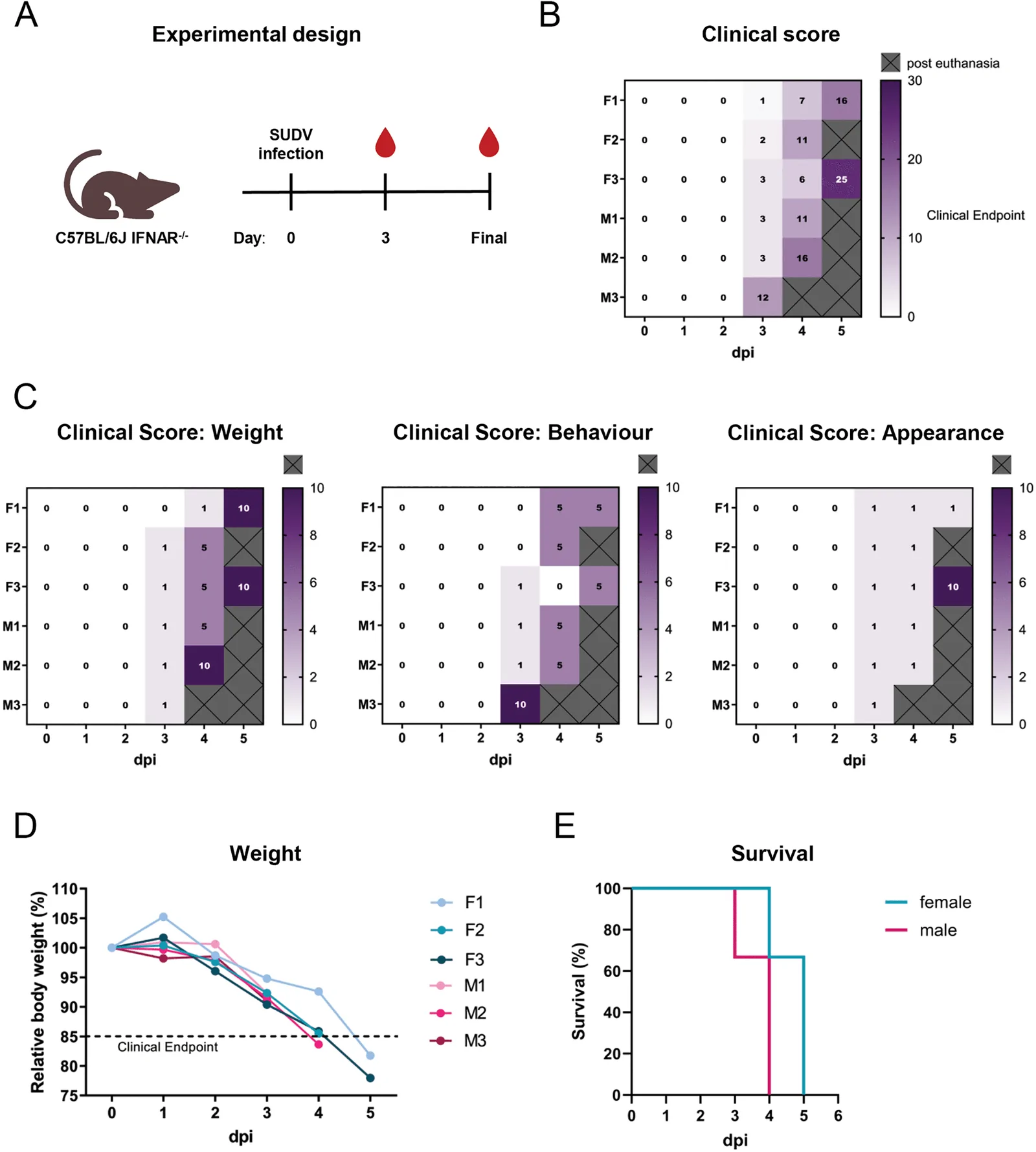
Clinical course of SUDV-infected IFNAR^-/-^ mice. The experimental approach is shown in **(A)**. Female (F) and male (M) IFNAR^-/-^ mice were infected with approx. 1,000 PFU SUDV on day 0 (n = 6) and monitored daily. The mice were euthanized at humane clinical endpoint (score of ≥10 on one day or ≥6 on two consecutive days). Blood samples were collected at day 3, and on the day of euthanasia. The additive clinical score **(B)**, summarizes the results of the different parameters analyzed daily: (C) body weight, behaviour, and appearance. Each parameter could be awarded a maximum of 10 points. Grey boxes indicate days after euthanasia. The relative body weight compared to day 0 for each mouse is depicted in **(D)**. The dotted line marks the clinical endpoint. The Kaplan-Meier survival curve **(E)** depicts the percentage of surviving mice over time.

Mice began exhibiting signs of disease from day 3 onwards, with the most commonly observed symptoms being progressive weight loss (6/6) and reduced self-grooming behaviour with ruffled fur (6/6), hypoactivity and reduced responsiveness (6/6) (Fig 1B and 1C). Additionally, more severe clinical manifestations included profound lethargy with complete unresponsiveness in one male mouse (M3), and haemorrhagic signs in one female mouse (F3), characterized by bleeding from nostrils and oral cavity on day 5.

All mice reached the predefined clinical endpoint, with clinical scores exceeding 10 as a result of the cumulative contribution of multiple clinical parameters. In most animals, the primary contributor was marked weight loss of more than 15% (10 points; Fig 1D), often accompanied by reduced responsiveness. In contrast, mouse M3 reached the humane endpoint primarily due to severe behavioural abnormalities, including profound lethargy and complete unresponsiveness to external stimuli, although a body weight loss of 9% was also observed. Overall, the SUDV mouse model was characterized by 100% lethality according to the pre-defined humane end points within 5 dpi (Fig 1E).

### The Sudan virus infection results in widespread organ dissemination and high viral loads

During the course of the study, blood samples were collected from the facial vein on day 3 as well as on the day of euthanasia. Viral loads were assessed using qRT-PCR and TCID_50_ from serum and organ homogenates (Fig 2). The qRT-PCR results are presented as original Ct values, with the y-axis inverted to facilitate interpretation, such that lower Ct values, corresponding to higher viral RNA concentrations, are displayed higher in the graph. As shown in Fig 2, substantial amounts of viral RNA were detected in all tested organs. The highest Ct values were found in the spleen (mean = 15,4), liver (mean = 17,1) and ovaries (mean = 16,39), while the lowest levels were found in the eyes (mean = 27,35) and brain samples (mean = 26,68). These values were relatively evenly distributed among the different organ samples, suggesting consistency of viral RNA detection across tissues within the analyzed animals. However, given the limited sample size, further studies with larger cohorts will be required to confirm the reproducibility and robustness of this model. In addition, potential effects of mouse age on disease progression, viral load distribution, and model reproducibility should be further investigated. qRT-PCR of serum samples showed similar values across all samples (mean = 23 on day 3), with the lowest results for F3 and M1 (dark green and light pink, respectively; Fig 2B). Missing values correspond to animals euthanized before the designated sampling timepoint.

**Fig 2:**
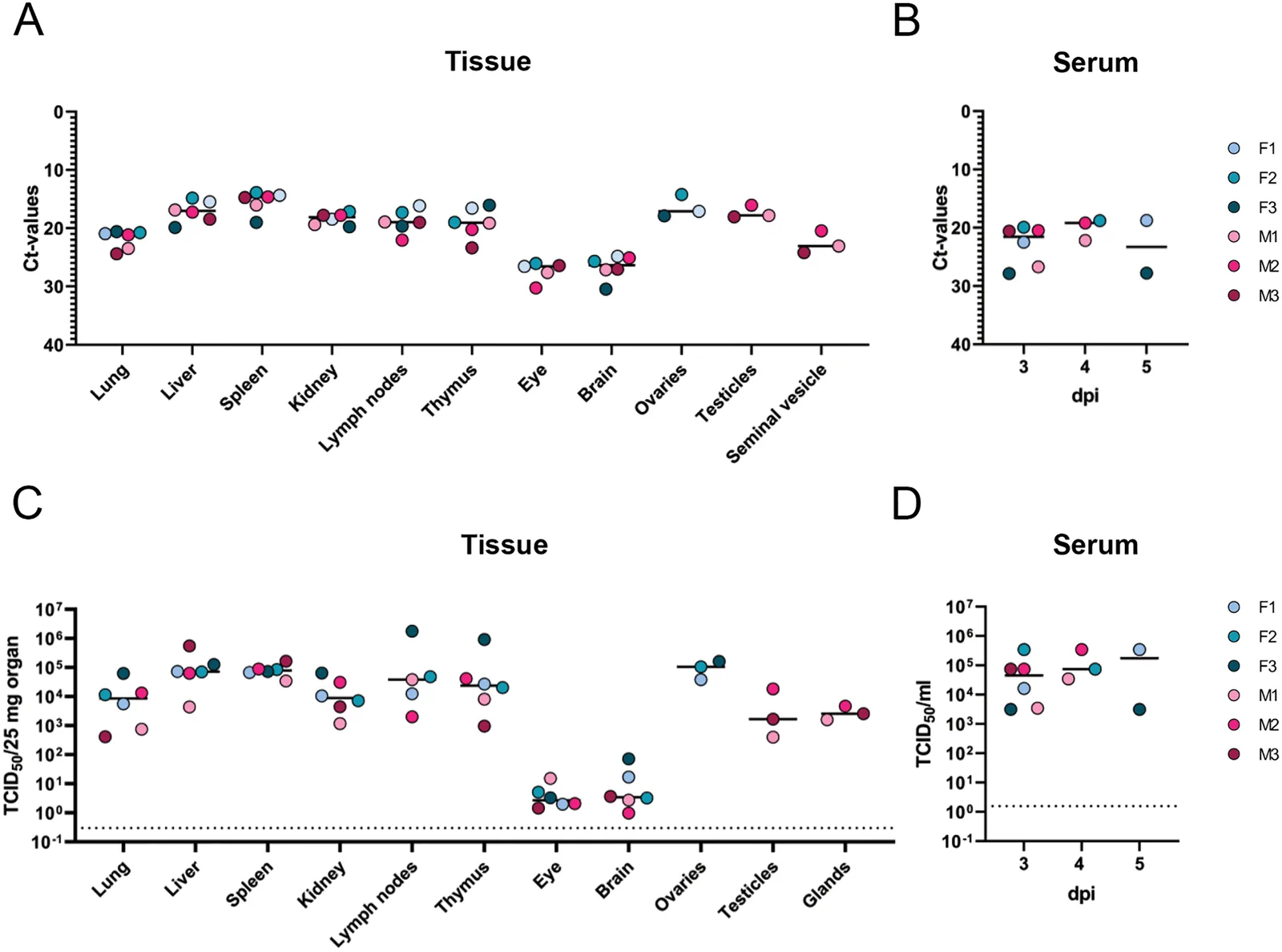
Detection of SUDV viral RNA and infectious titers in infected IFNAR^-/-^ mice. Blood samples were collected on day 3 and on the day of euthanasia, together with samples of different organs. The amounts of SUDV viral RNA were measured by RT-qPCR in the organs **(A)** and serum samples **(B)** of individual mice. The qRT-PCR results are presented as original Ct values, with the y-axis inverted to facilitate interpretation. Infectious SUDV titers were determined by TCID_50_ assay in organs **(C)** and serum samples **(D)** of individual mice. The titers present in the organs were normalised to their weight. Data are shown as mean with individual values. Dotted lines indicate the LOD (see the Methods section for details). **F:** female; **M:** male.

Infectious virus was also detected in all organs (Fig 2C) and in all serum samples (Fig 2D), confirming systemic infection. However, in the case of mouse M3, the titer for the lymph nodes was below the LOD due to an unspecific toxic effect on cells in the first dilution. Re-titration using other dilution schemes (1:2 and 1:5 instead of 1:10) yielded no improved result. The organs with the highest titers included liver (mean = 1,5x10^5^ TCID_50_/25 mg organ), spleen (mean = 8,6x10^4^ TCID_50_/25 mg organ), and ovaries (mean = 1x10^5^ TCID_50_/25 mg organ). On day 3, the average serum titer was 8.6x10^4^ TCID_50_/ml. This increased further for surviving animals at 4 and 5 dpi (mean = 1.6x10^5^). Overall, the qRT-PCR results are consistent with the TCID_50_ values, indicating high viral RNA loads and infectious particles in all organs. This suggests that all tested organs supported productive infection.

Moreover, the harvested organs were analyzed histopathologically using H&E staining. Of the organs evaluated, the liver and spleen were the most affected and showed marked evidence of tissue damage. In the liver (Fig 3A-3D and S1 Fig), randomly distributed multifocal hepatocellular necrosis was evident (dotted lines), characterized by hepatocytes with hypereosinophilic cytoplasm (filled arrows, dark blue) and pyknotic or karyorrhectic nuclei (filled arrowheads, dark blue). In addition, variable and partially severe macrovesicular (and/or microvesicular) vacuolation of hepatocytes was noted (hollow arrows, dark blue), accompanied by mixed inflammatory-cell infiltration (hollow arrowheads). In the spleen (Fig 3E-3H and S1 Fig), the white pulp exhibited marked lymphoid depletion, (filled arrows, dark grey), while the red pulp contained foci with necrotic cells (filled arrowheads, dark grey) and patchy infiltration with mononuclear cells (sinus histiocytosis, hollow arrowheads, dark grey). Tissue damage was evaluated semi-quantitatively and scored from 0–3, with a score of 3 indicating greater severity. The highest scores for the liver and spleen were observed in mice F1 and F3, which survived the longest, whereas mouse M3, which were euthanized on day 3, showed the lowest score for both organs. This pattern suggests a possible association between disease duration and the severity of pathology, with mice surviving longer showing a more time-dependent progression of tissue damage. However, this observation should be interpreted cautiously, as it may also reflect differences in individual disease trajectories. Confirmation in a follow-up study using larger animal cohorts and predefined euthanasia time points will be necessary to substantiate this relationship and better define the temporal dynamics of lesion development.

**Fig 3:**
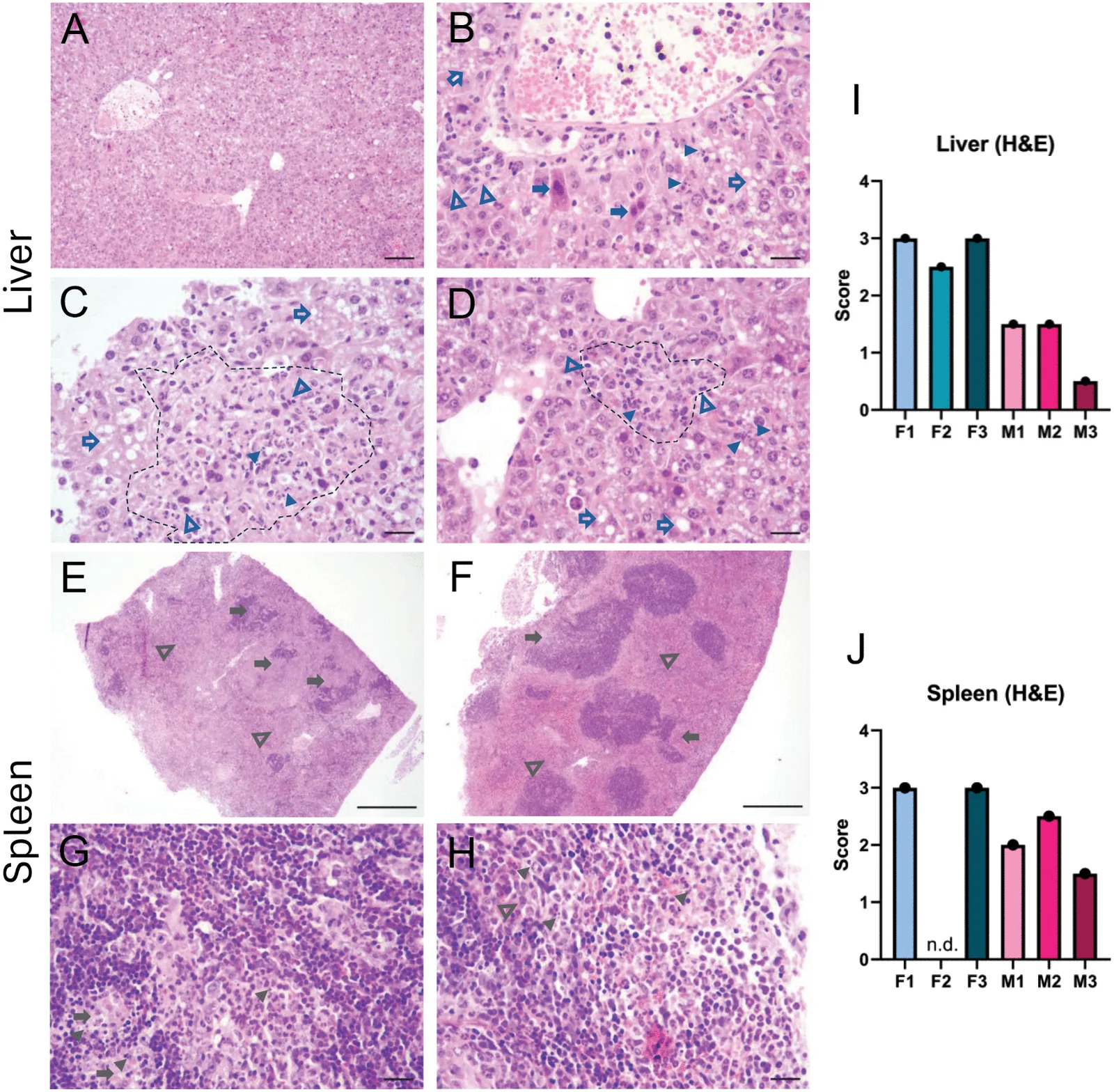
Pathology of SUDV-infected IFNAR^-/-^ mice. Organs were harvested after euthanization and pathologically examined *post mortem*. Exemplary images of liver **(A-D)** and spleen **(E-H)** stained with H&E are shown. In livers, multifocal liver necrosis, eosinophilic hepatocytes, lymphohistiocytic infiltrates and vacuolation were observed. In spleens, lymphoid depletion, necrotic cells as well as mononuclear infiltrates were observed. For each animal, scores for disease progression were determined (**I and J** for liver and spleen, respectively). Scale bars: A: 100 µm; B-D, G-H: 25 µm; E-F: 500 µm. **H&E:** hematoxylin and eosin; **n.d.:** no data; **F:** female; **M:** male.

The presence of genomic SUDV RNA in harvested organs was assessed via *in situ* hybridization using the V-SudanEbola-NP-sense probe, which hybridizes to antisense RNA. Red staining indicative of SUDV RNA was detected in multiple organs, with the most prominent staining observed in liver and spleen (Fig 4A-4D). In these tissues, SUDV RNA appeared in small, scattered foci throughout the parenchyma, although the extent varied among individual mice. Notably, M1 and M2 exhibited strong staining in both liver and spleen, whereas M3 and F2 displayed only limited signal. In the brain, discrete foci positive for viral RNA were observed in the cortex, meninges and the choroid plexus (Fig 4E-4F). Occasionally, signals for viral RNA were also detected in the lungs (Fig 4G-4H), kidneys, visceral fat tissue, and testes. While overall detection of viral RNA levels was low, it was sufficient to be associated with severe tissue damage, which was likely driven by elevated and uncontrolled inflammatory responses. Interestingly, mice with the highest ISH RNA signal received the lowest scores for tissue damage (S2 Fig and S3 Table). A possible inverse relationship between ISH RNA signal and tissue damage was therefore observed. Together with the finding that the most extensive lesions were present in mice surviving the longest, this observation may suggest that detectable viral RNA levels decrease during disease progression. It is possible that advanced tissue damage and/or late-stage disease with resulting inflammatory processes may affect the persistence or detectability of viral RNA within affected tissue.

**Fig 4:**
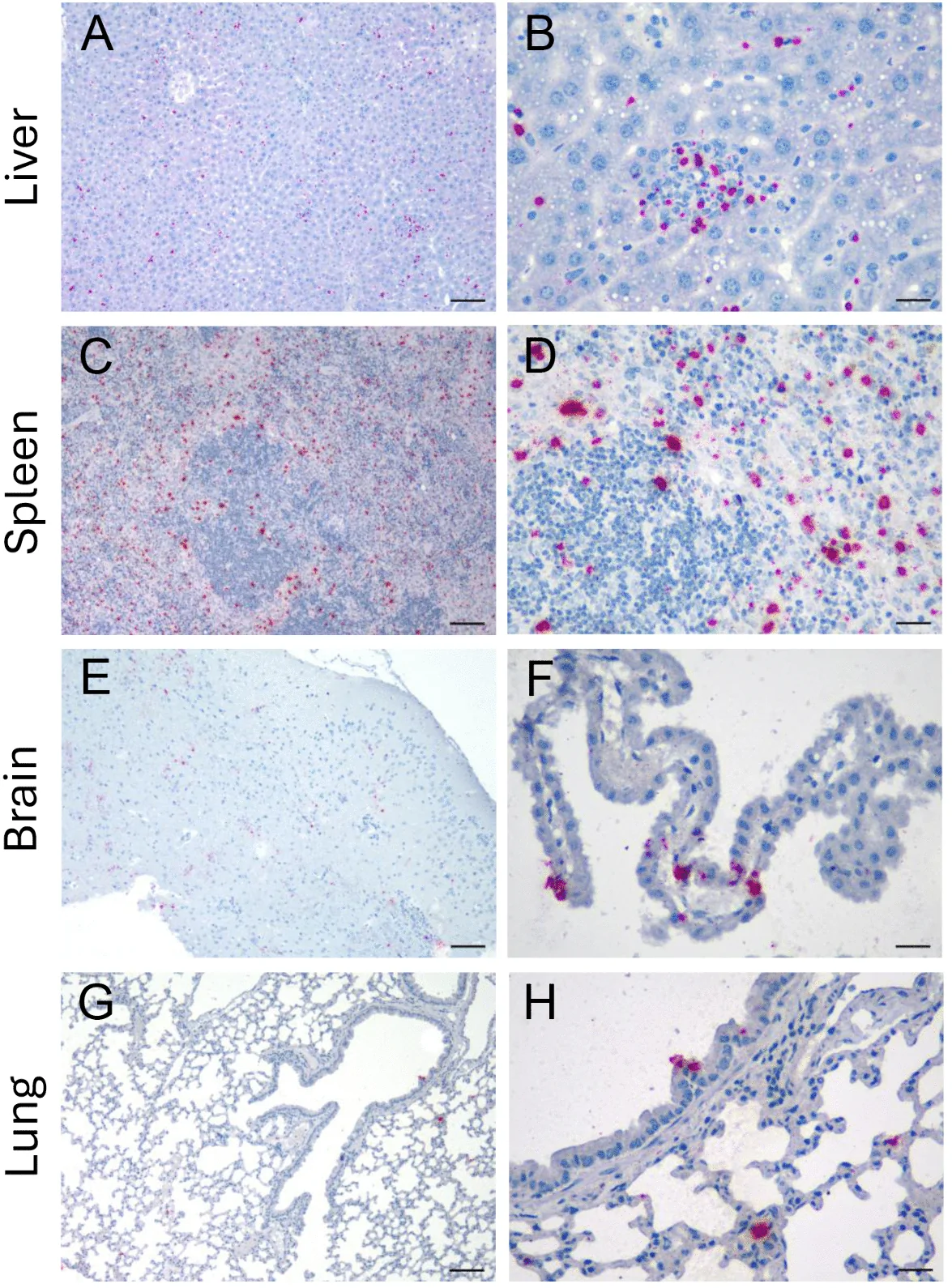
Detection of SUDV viral RNA in organs of IFNAR^-/-^ mice by *in situ* hybridization. Organs were harvested after euthanization and pathologically examined *post mortem*. Exemplary images of liver **(A-B)**, spleen **(C-D)**, brain **(E-F)**, and lungs **(G-H)** stained for viral genomic RNA (V-SudanEbola-NP-sense) by ISH are shown (red staining). Scale bars: Left panel 100 µm; right panel 25 µm. ISH: *in situ* Hybridization.

In summary, these results support prior reports showing IFNAR^-/-^ mice to be a suitable model for SUDV infections [4,7,8,13–16].

#### Sudan virus infection induces broadly elevated cytokine and chemokine responses.

Fatal filovirus infections are characterized by immune suppression and dysregulated inflammatory responses [25,26]. To investigate whether this pattern is recapitulated in our model, we performed a chemokine/cytokine analysis and tested different pro-inflammatory cytokines, regulatory and anti-inflammatory cytokines, T-cell differentiation-associated cytokines, hematopoietic growth factors and chemokines for chemotaxis and recruitment, summarized in S2 Table [27].

As shown in Fig 5, all analytes in our panel were significantly upregulated in the SUDV-infected mice compared to our historical uninfected controls. Mouse F3 was not included in this analysis due to insufficient serum volume. A trend was observed in which male mice exhibited higher concentrations of multiple mediators than female mice under both infected and control conditions. However, the small sample size limits the interpretation of these apparent differences. Moreover, mediators associated with different types of immune responses were significantly increased, including IFN-γ and IL-12 driving type I immunity; IL-4, IL-5, and IL-13 driving type II immunity; and IL-17a driving type III immunity. This simultaneous activation indicates a dysregulated immune response that can weaken or misdirect the type I immunity – which is crucial for clearing viral infections – ultimately impairing viral clearance and promoting pathological inflammation [28].

**Fig 5:**
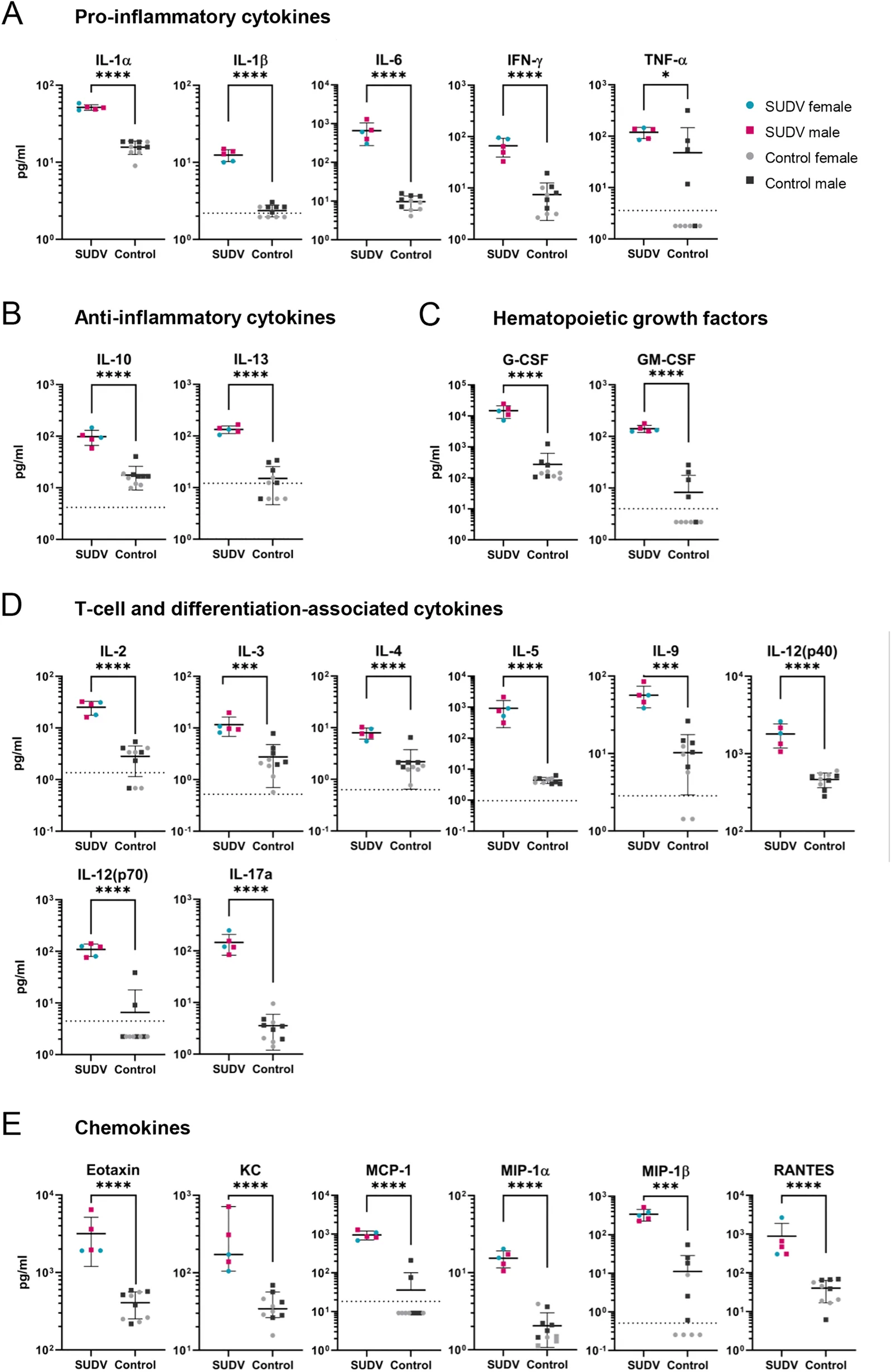
Systemic cytokine and chemokine response in SUDV-infected IFNAR^-/-^ mice. The presence of 23 mediators in the final serum samples of SUDV-infected mice and uninfected controls (n = 10) was analyzed using multiplex ELISA based on the Luminex technology. Data are presented as mean ± SD, with individual values shown. Dotted lines indicate the lowest standard, defined as LLOQ. The data were log transformed for normal distribution prior to statistical analysis. The *p*-values are shown above each graph and refer to unpaired t-tests (see the Methods section for details). **ELISA:** enzyme-linked immunosorbent assay; **SD:** standard deviation; **LLOQ:** lower limit of quantification.

## Discussion

Here, we characterized a lethal SUDV Boniface infection in C57BL/6J IFNAR^-/-^ mice, with animals reaching the clinical endpoint between day 3–5 after infection and being sacrificed with clinical scores of at least 10. The mice in the present study were sacrificed earlier than in other studies due to stricter humane endpoints. Therefore, we cannot exclude the possibility that some animals may have stabilized or recovered if less stringent endpoint criteria had been applied. Reported outcomes vary widely across models: Brannan et al. (2015) and Furuyama et al. (2016) found high survival in WT and IFNAR^-/-^ mice infected with SUDV Gulu or Boniface, whereas Froude et al. (2018), Comer et al. (2019), Escaffre et al. (2021), and Flaxman et al. (2024) reported uniformly lethal infections with varying disease onset and weight loss. Escudero-Pérez et al. (2019) observed ~71% lethality in humanized mice, and Lever et al. (2012) reported non-lethal infection with ~15% weight loss after airborne infection [7,8,13–17,29]. Overall, these differences highlight that disease severity and lethality in SUDV mouse models are not fixed properties of the virus–host interaction alone, but instead reflect a combination of viral isolate-specific characteristics, experimental design variables, and study-dependent humane endpoint criteria, which together can substantially influence the observed clinical outcome.

Overall, SUDV Gulu and Boniface cause severe disease and high mortality, although outcomes vary depending on euthanasia criteria, mouse strain, and virus isolate. Only one study has directly compared the two SUDV strains, suggesting a potentially higher virulence of SUDV Boniface, although additional comparative studies are needed to confirm this observation [8].

SUDV infection resulted in high viral RNA loads and infectious particles in serum and all analyzed tissues, indicating widespread systemic viral dissemination. The highest loads were detected in the liver and spleen, followed by lymph nodes and thymus, consistent with previous reports of filovirus infection in humans showing prominent viral replication in secondary lymphoid organs, spleen, and liver [26]. As has been reported for human patients infected with SUDV, fatal cases correlate with high viral RNA loads [30,31], which has been used as a predictor of outcome. This is also reflected in our model, where we observed uniform lethality and high viral loads. Notably, viral RNA was also detected in reproductive organs (ovaries, testes, and accessory glands). As reports of viral persistence and sexual transmission have increased [32], these findings may be relevant but were not specifically investigated in the present study and therefore require further investigation. A mouse model for Ebola virus (EBOV) sexual transmission recently developed by Clancy and colleagues [33] may provide insights.

Histopathology revealed severe hepatosplenic damage. However, ISH staining for viral RNA was overall less extensive compared to EBOV-infected IFNAR^-/-^ mice [10]. Similarly, Ellis and colleagues [34] found EBOV caused more widespread lesions and higher viral loads than SUDV in rhesus monkeys.

Variation in ISH staining among mice potentially reflects differences in euthanasia timing as mice euthanized later (M1 and M2, day 4) showed the highest loads, while one mouse euthanized earlier (M3, day 3) showed the lowest levels. These observations highlight the potential influence of disease stage and euthanasia timing on viral distribution and pathology.

Slight sex-dependent differences were observed. For instance, male mice reached the humane endpoint on average one day earlier than female mice, and showed, in some instances, higher serum cytokine and chemokine concentrations. However, due to the limited number of animals, no definitive conclusions can be drawn. Potential confounding variables, such as differences in baseline body weight and the timing of euthanasia, should also be considered when interpreting these findings. Further studies with larger cohorts will be required to clarify these observations. Nevertheless, sex-specific differences in basal cytokine and chemokine levels, as well as in responses to noxious stimuli including infection, injury, or inflammation, have been described previously [35–38]. Male mice have often been reported to exhibit stronger pro-inflammatory cytokine responses (e.g., IL-6, TNF-α) to infection or injury, which may be associated with more pronounced sickness behavior. Conversely, female mice have been reported to display higher levels of specific chemokines that enhance leukocyte recruitment and immune cell reactivity, potentially contributing to more effective, but in some circumstances more pronounced, immune responses.

Fatal filovirus infections of humans are characterized by immune suppression, uncontrolled inflammation, and weak humoral responses, whereas survivors show early and moderate immune activation with detectable IgG and IgM [25,26,39]. Infected macrophages and monocytes release cytokines and chemokines such as TNF-α, IL-1β, IL-6, IL-8, IL-10, MCP-1, MIP-1α, MIP-1β, and IP-10, driving a dysregulated immune response, termed “cytokine storm” that can cause systemic damage and multi-organ failure [39–41]. Fatal cases also show suppressed IFN-α, elevated pro-inflammatory mediators, and loss of CD4 ⁺ /CD8 ⁺ T cells with reduced T-cell cytokines [42,43]. Especially elevated levels of IL-6, IL-10 and MIP-1β have been specifically shown by Hutchinson and Rollin in 2007 and McElroy et al. for patients infected with SUDV in 2000/2001, using a similar Luminex technology as in the present study [31,44]. Consistent with these findings, the SUDV-infected mice of this study exhibited high serum cytokine and chemokine levels together with pronounced hepatosplenic pathology, suggesting a robust systemic inflammatory response that has not previously been described in such detail for SUDV infection in mice [27]. However, these findings should be interpreted with caution, as a significant limitation of this study is that cytokine and chemokine analyses were performed only on terminal samples.

Escudero-Pérez et al. compared EBOV and Reston virus (RESTV) infection in humanized mice using Luminex cytokine profiling. Serum data at 3 and 6 dpi are most comparable to our study. Relative to their EBOV data, the IFNAR^-/-^ mice in the present study showed similar IFN-γ and IL-10 levels, higher IL-6, RANTES, and MCP-1, but lower MIP-1α (S4 Table). In RESTV-infected mice, cytokine levels were similar or slightly lower than in EBOV infections, with markedly higher levels in non-survivors. EBOV cytokines generally rose over time, while RESTV levels stabilized or declined. If technical and strain differences between the two studies are neglected, cytokine expression ranked roughly RESTV < SUDV < EBOV [17].

Luminex analysis of BALB/c mice infected with mouse-adapted Marburg virus (MARV) Angola showed significantly higher cytokine and chemokine levels than the symptomless wild-type strain at 6 dpi [45], though the magnitude of induction differed markedly from our SUDV results (S4 Table).

In Cynomolgus macaques, SUDV infection triggered strong cytokine and chemokine responses, broadly consistent with those observed in our mouse model [46]. Notably, TNF-α, IFN-γ, and IL-2 – unchanged in human SUDV cases – were nonetheless elevated in both macaque studies and in our data, though to varying degrees. A second non-human primate study reported similar overall trends, with only modest differences in magnitude across individual analytes [47].

Luminex analysis of SUDV (Gulu)-infected human patients showed significantly elevated IL-1β, IL-6, IL-10, IP-10, MIP-1β, and RANTES, whereas TNF-α, IL-2, and IFN-γ were not upregulated compared to healthy controls [44]. This contrasts with our SUDV study and EBOV reports [25,42], although TNF-α was the least affected parameter in our study (Fig 5). Another study of the 2000–2001 Uganda outbreak found increased IL-1α, IL-6, MCP-1, and MIP-1α in fatal cases, consistent with our results, while IL-8 (KC homolog) rose only at late infection stages [31]. Elevated M-CSF, MIP-1α, and IP-10 were linked to hemorrhages, whereas TNF-α was not significant and IL-2 and IFN-γ were excluded from analysis [31].

S4 Table summarizes cytokine and chemokine regulation across species.

The increase of TNF-α, IFN-γ, and IL-2 in macaques and our mouse model, but not in humans, raises questions about their role in SUDV pathogenesis, especially given their known upregulation in EBOV infection. However, controlled animal studies allow for precise timing of sample collection, whereas human samples are typically obtained at variable and often late stages of disease. These differences in sampling time may obscure smaller but biologically meaningful cytokine changes in human infections, especially because TNF-α is mainly present in the acute phase of infection and decays quickly [42].

IFNAR^-/-^ and similar knockout (KO) mice are often a subject of critique as their immune system is different from WT mice which raises the questions of whether KO mice are indeed suitable models to study filovirus infection. A feasible attempt to avoid both KO mice and mouse-adapted viruses is the treatment of WT mice with IFN antibodies prior to infection to enable/enhance susceptibility [4]. However, similarities in chemokine and cytokine profiles reported across different mouse models, as well as between mice, NHPs, and humans, suggest that selected features of the inflammatory response may be conserved across experimental systems.

In summary, we present a characterization of SUDV Boniface infection in IFNAR^-/-^ mice. Under the conditions used, the model produced a uniformly lethal disease characterized by systemic viral dissemination, hepatosplenic pathology, and elevated terminal cytokine and chemokine concentrations. These findings are consistent with typical features of filovirus infection and support further evaluation of the IFNAR^-/-^ mouse as a model for SUDV infection and for preclinical studies of filovirus countermeasures. However, the limited cohort size, single experimental study, and terminal-only assessment of cytokines limit conclusions regarding the temporal development and mechanistic basis of the inflammatory response.

## Supporting information

S1 Table Evaluation table of clinical scores during challenge experiments. (DOCX)

S2 Table Investigated cytokines and chemokines using the Luminex technology. (DOCX)

S3 Table Comparative ranking of results across all evaluated parameters. qPCR, TCID₅₀, and histopathological results for each mouse were ranked from 1 to 6, with 1 representing the highest measured value. qPCR data are presented as 40 − Ct values. Histological assessment of liver and spleen tissue was performed using H&E staining and ISH. Tissue injury and viral RNA abundance were scored semi-quantitatively on a scale of 0–3, with 3 denoting the most severe findings. F: female; M: male; dpi: days post infection; LOD: limit of detection.
(DOCX)

S1 Fig Haematoxylin and eosin staining of liver and spleen in SUDV-infected IFNAR^-/-^ mice. Organs were harvested after euthanization and pathologically examined post mortem. Exemplary images of liver and spleen from all mice stained with H&E are shown. Scale bars: 500 µm. IFNAR^-/-^: Interferon receptor alpha/beta knockout mice; F: female; M: male; H&E: Haematoxylin and eosin.
(JPG)

S2 Fig Detection of SUDV viral RNA in liver and spleen of IFNAR^-/-^ mice by *in situ* Hybridization. Organs were harvested after euthanization and pathologically examined post mortem. Exemplary images of liver and spleen from all mice stained for viral genomic RNA (V-SudanEbola-NP-sense) via ISH are shown. Scale bars: panel 1 and 3, 100 µm; panel 2 and 4, 25 µm. SUDV: Sudan virus; IFNAR^-/-^: Interferon receptor alpha/beta knockout mice; PFU: plaque forming units; ISH: *in situ* Hybridization; F: female; M: male.
(JPG)

S4 Table Comparative analysis inflammation markers in mice after filovirus infection using the Luminex technology. Values for other publications are approximates/approximate averages. Mean cytokine and chemokine concentrations were estimated by visual inspection of (graphical) data in cited publications (including their supplemental material). Values do not necessarily reflect the exact original measurement but rather should be regarded as approximates and are intended to illustrate relative trends and enable contextual comparisons. Original published (numerical) data, where available, should be considered authoritative. n.s. no upregulation compared to (noninfected) controls. # Reported as comparable to controls; numerical values were not provided.
(DOCX)

## Abbreviations

BSL-4: biosafety level-4
dpi: days post infection
EBOV: Ebola virus
ELISA: enzyme-linked immunosorbent assay
G-CSF: granulocyte-colony stimulating factor
GM-CSF: granulocyte-macrophage colony-stimulating factor
IFN-γ: interferon gamma
IFNAR^-/-^: interferon receptor alpha/beta knockout mice
IL: interleukin
i.p.: intraperitoneally
KC: keratinocyte-derived chemokine
LLOQ: lower limit of quantification
LOD: limit of detection
MARV: Marburg virus
MCP-1: monocyte chemotactic protein-1
MIP: macrophage inflammatory protein
PFU: plaque forming units
RANTES: Regulated upon Activation, Normal T cell Expressed and Secreted
RESTV: Reston virus
SUDV: Sudan virus
TNF-α: tumor necrosis factor alpha.
