# Supplementary material for: Lethal Sudan virus infection in IFNAR-/- mice is characterized by key inflammatory features of filovirus disease
Source: PLoS One. 2026 Sep 29;21(9):e0347080. doi: 10.1371/journal.pone.0347080 (PMC13623132; doi:10.1371/journal.pone.0347080)
Supplement: Supplementary file 1 [file pone.0347080.s001.docx]

**S1 Table: Evaluation table of clinical scores during challenge experiments**

| **Scoring** | **0** | **1** | **5** | **10** |
| --- | --- | --- | --- | --- |
| **Body weight (deviation from average individual weight*)** | 0% – 5% | 6% – 10% | 11% – 15% | > 15% |
| **General condition** | Fur smooth, shiny; Orifices clean; Eyes clear | Fur slightly ruffled, poor or excessive grooming | Fur dull, disordered; unkempt orifices; Eyes cloudy | Focally sticky or shaggy, rough fur; Eyes cloudy; Diarrhea; Jaundice or evidence of coagulopathy |
| **Spontaneous behaviour** | Normal behaviour (sleeping, reacting to touch, curiosity, social contact) | Minor deviations from normal behaviours | Moderate changes in activity (hyperactivity/ hypoactivity) | Severe activity changes or lethargy; Self- isolation, aggressive behaviour towards oneself or cage mates |
| * The score in the three different categories (body weight, general condition and spontaneous behaviour) for each animal is summed up and animals were euthanized when they reached a score of 10, i.e. the evaluation in one of the criteria body weight, general condition and spontaneous behaviour with a score of 10 was sufficient for euthanasia. If, by adding up the results of the various criteria, a score of 10 on a single day or 6 on two consecutive days was obtained, the veterinarian was consulted to decide on the euthanasia. | | | | |
