# Supplementary material for: Lethal Sudan virus infection in IFNAR-/- mice is characterized by key inflammatory features of filovirus disease
Source: PLoS One. 2026 Sep 29;21(9):e0347080. doi: 10.1371/journal.pone.0347080 (PMC13623132; doi:10.1371/journal.pone.0347080)
Supplement: Supplementary file 2 [file pone.0347080.s002.docx]

**S3 Table: Investigated cytokines and chemokines using the Luminex technology.**

| **Functional Category** | **Cytokines / Chemokines** | **Primary Biological Role** |
| --- | --- | --- |
| Pro-inflammatory cytokines | IL-1α, IL-1β, IL-6, IFN-γ, TNF-α | Initiation and amplification of inflammatory responses; activation of innate immune pathways and promotion of immune cell effector functions |
| Regulatory and anti-inflammatory cytokines | IL-10, IL-13 | Modulation of immune activity; limitation of excessive inflammation and promotion of immune homeostasis |
| T-cell differentiation-associated cytokines | IL-2, IL-3, IL-4, IL-5, IL-9, IL-12 (p40), IL-12 (p70), IL-13, IL-17A | Regulation of T-cell activation, proliferation, and polarization; coordination of innate and adaptive immune responses |
| Hematopoietic growth factors | G-CSF, GM-CSF | Stimulation of granulocyte and monocyte production and activation; support of the myeloid immune response |
| Chemokines (chemotaxis and recruitment) | Eotaxin, KC, MCP-1, MIP-1α, MIP-1β, RANTES | Chemotactic recruitment of leukocyte subsets to sites of inflammation and tissue injury |
