# Supplementary material for: Lethal Sudan virus infection in IFNAR-/- mice is characterized by key inflammatory features of filovirus disease
Source: PLoS One. 2026 Sep 29;21(9):e0347080. doi: 10.1371/journal.pone.0347080 (PMC13623132; doi:10.1371/journal.pone.0347080)
Supplement: Supplementary file 3 [file pone.0347080.s003.docx]

**S2 Table. Comparative ranking of results across all evaluated parameters.**

qPCR, TCID₅₀, and histopathological results for each mouse were ranked from 1 to 6, with 1 representing the highest measured value. qPCR data are presented as 40 − Ct values. Histological assessment of liver and spleen tissue was performed using H&E staining and ISH. Tissue injury and viral RNA abundance were scored semi-quantitatively on a scale of 0–3, with 3 denoting the most severe findings. **F:** female; **M:** male; **dpi:** days post infection; **LOD:** limit of detection.

| **Mouse** | | **F1** | **F2** | **F3** | **M1** | **M2** | **M3** |  | **Ranking** |
| --- | --- | --- | --- | --- | --- | --- | --- | --- | --- |
| Euthanasia (dpi) | | 5 | 4 | 5 | 4 | 4 | 3 |  | 1 |
| 40-Ct | Lung | 19,1 | 19,3 | 19,4 | 16,6 | 18,9 | 15,6 |  | 2 |
|  | Liver | 24,5 | 25,2 | 20,2 | 23,2 | 22,8 | 21,6 |  | 3 |
|  | Spleen | 25,6 | 26,1 | 21,0 | 24,0 | 25,4 | 25,3 |  | 4 |
|  | Kidney | 21,6 | 22,9 | 20,3 | 20,6 | 22,2 | 22,2 |  | 5 |
|  | Lymph nodes | 23,9 | 22,7 | 20,4 | 21,1 | 18,0 | 21,0 |  | 6 |
|  | Thymus | 23,4 | 21,0 | 24,0 | 20,9 | 19,8 | 16,7 |  |  |
|  | Eye | 13,5 | 14,0 | <LOD | 12,4 | 9,8 | 13,6 |  |  |
|  | Brain | 15,2 | 14,4 | 9,6 | 12,9 | 14,9 | 13,0 |  |  |
|  | Ovaries | 22,9 | 25,8 | 22,1 |  |  |  |  |  |
|  | Testicles |  |  |  | 22,2 | 24,0 | 22,0 |  |  |
|  | Glands |  |  |  | 17,0 | 19,6 | 15,9 |  |  |
|  | Average  ranking | 2 | 1 | 6 | 4 | 3 | 5 |  |  |
| TCID_50_/ml | Lung | 5,6E+03 | 1,2E+04 | 6,3E+04 | 7,5E+02 | 1,3E+04 | 4,1E+02 |  |  |
|  | Liver | 7,3E+04 | 7,1E+04 | 1,3E+05 | 4,4E+03 | 6,3E+04 | 5,6E+05 |  |  |
|  | Spleen | 6,8E+04 | 8,6E+04 | 7,3E+04 | 3,4E+04 | 8,9E+04 | 1,6E+05 |  |  |
|  | Kidney | 1,1E+04 | 7,2E+03 | 6,5E+04 | 1,2E+03 | 3,1E+04 | 4,5E+03 |  |  |
|  | Lymph nodes | 1,3E+04 | 4,8E+04 | 1,8E+06 | 3,8E+04 | 2,0E+03 | *<LOD* |  |  |
|  | Thymus | 2,7E+04 | 2,1E+04 | 9,3E+05 | 8,1E+03 | 4,2E+04 | 9,7E+02 |  |  |
|  | Eye | 2,0E+00 | 5,1E+00 | 3,3E+00 | 1,5E+01 | 2,1E+00 | 1,5E+00 |  |  |
|  | Brain | 1,7E+01 | 3,3E+00 | 7,2E+01 | 2,7E+00 | 9,7E-01 | 3,7E+00 |  |  |
|  | Ovaries | 3,9E+04 | 1,1E+05 | 1,6E+05 |  |  |  |  |  |
|  | Testicles |  |  |  | 4,0E+02 | 1,8E+04 | 1,7E+03 |  |  |
|  | Glands |  |  |  | 1,6E+03 | 4,7E+03 | 2,6E+03 |  |  |
|  | Average  ranking | 4 | 3 | 1 | 6 | 2 | 5 |  |  |
| Serum  Ct-40 | 3 dpi | 17,52 | 20,07 | 12,16 | 13,28 | 19,5 | 19,41 |  |  |
|  | 4 dpi |  | 21,23 |  | 17,83 | 20,83 |  |  |  |
|  | 5 dpi | 21,25 |  | 12,21 |  |  |  |  |  |
| Serum TCID_50_/ml | 3 dpi | 1,61E+04 | 3,46E+05 | 3,15E+03 | 3,46E+03 | 7,45E+04 | 7,45E+04 |  |  |
|  | 4 dpi |  | 7,45E+04 |  | 3,46E+04 | 3,46E+05 |  |  |  |
|  | 5 dpi | 3,46E+05 |  | 3,15E+03 |  |  |  |  |  |
| Histology | Liver (H&E) | 3 | 2,5 | 3 | 1,5 | 1,5 | 0,5 |  |  |
|  | Spleen (H&E) | 3 |  | 3 | 2 | 2,5 | 1,5 |  |  |
|  | Liver (ISH) | 1 | 0,5 | 0 | 2 | 1,5 | 0,5 |  |  |
|  | Spleen (ISH) | 2 |  | 1 | 3 | 3 | 0,5 |  |  |
