# Supplementary material for: Lethal Sudan virus infection in IFNAR-/- mice is characterized by key inflammatory features of filovirus disease
Source: PLoS One. 2026 Sep 29;21(9):e0347080. doi: 10.1371/journal.pone.0347080 (PMC13623132; doi:10.1371/journal.pone.0347080)
Supplement: Supplementary file 4 [file pone.0347080.s006.docx]

**S4 Table. Comparative analysis inflammation markers in mice after filovirus infection using the Luminex technology.**

Values for other publications are approximates/approximate averages. Mean cytokine and chemokine concentrations were estimated by visual inspection of (graphical) data in cited publications (including their supplemental material). Values do not necessarily reflect the exact original measurement but rather should be regarded as approximates and are intended to illustrate relative trends and enable contextual comparisons. Original published (numerical) data, where available, should be considered authoritative. n.s. no upregulation compared to (noninfected) controls. # Reported as comparable to controls; numerical values were not provided.

| **Publication** | **Gellhorn Serra et al. 2026** | **Escudero-Pérez et al. 2019** | **Qiu et al.**  **2014** | **Alfson et al.**  **2022** | **Chan et al.**  **2024** | **Fletcher et al. 2025** | **Hutchinson und Rollin 2007** | **McElroy et al.**  **2014** |
| --- | --- | --- | --- | --- | --- | --- | --- | --- |
| Organism | Mice (IFNAR^-/-^ C57BL/6J) | Mice (Humanized) | Mice (BALB/c) | NHPs | NHPs | NHPs | humans | humans |
| Virus | SUDV Boniface | EBOV | mouse-adapted MARV Angola | SUDV Gulu | SUDV Boniface | SUDV Gulu | SUDV Gulu | SUDV Gulu |
| **Analyte** | **Mean concentration [pg/ml]** | | | | | | | |
| **TNFα** | **119** |  | **150** | **100** | **1,000** | **40** | **100 (**n.s.**)** |  |
| **IFN-γ** | **66** | **200** | **1,000** | **100** | **1,000** | **400** | **100 (**n.s.**)** |  |
| IL-1β | 12 | <100 | 400 | 10 | 100 | 6 | 10 |  |
| IL-2 | 25 |  | 70 | 1 | 1,000 |  | # |  |
| IL-4 | 8 |  | 1,000 | 1 | 1,000 | 3 | # |  |
| IL-5 | 926 |  | 700 | >100 |  |  |  |  |
| IL-6 | 658 | 250 | 150 | 1000 |  | 2,8 | 1,000 | 2,000 |
| **IL-10** | **98** | **250** | **300** | **100** | **1** | **30** | **1,000** |  |
| IL-13 | 134 |  | 60 | 10 |  |  |  |  |
| IL-17a | 146 |  | 0,4 | 1 | 1,000 |  |  |  |
| MIP-1α | 15 | 200 | 1,000 | 1 | 1,000 |  | # | 30 |
| MIP-1β | 342 |  |  | 10 | 10,000 |  | ≤10,000 |  |
| IP-10 |  |  |  |  | 1,000 |  | ≤100 | 20,000 |
| RANTES | 880 | 350 |  |  | 10,000 |  | 10,000 |  |
| IL-8/KC | 288 | 400 | 400 | 1 | 100 |  | <10,000 | # but elevated |
| GM-CSF | 141 |  |  | 10 | 1,000 | 8 | # |  |
| MCP-1 | 951 | 200 | 15,000 | 1 |  | 20,000 |  | 500 |
